# Salivary glands harbor more diverse microbial communities than gut in *Anopheles culicifacies*

**DOI:** 10.1186/1756-3305-7-235

**Published:** 2014-05-20

**Authors:** Punita Sharma, Swati Sharma, Rakesh Kumar Maurya, Tanwee Das De, Tina Thomas, Suman Lata, Namita Singh, Kailash Chand Pandey, Neena Valecha, Rajnikant Dixit

**Affiliations:** 1 Host-Parasite Interaction Biology Group, National Institute of Malaria Research, Sector-8, Dwarka, Delhi 110077, India; 2 Department of Bio & Nano Technology, Guru Jambheswar University of Science and Technology, Hisar, Haryana 125001, India; 3 NxGenBio Life Sciences, C-451, Yojna Vihar, Delhi 110092, India

## Abstract

**Background:**

In recent years, it has been well documented that gut flora not only influence mosquito physiology, but also significantly alter vector competency. Although, salivary gland and gut constitute key partners of the digestive system, it is still believed that salivary glands may harbor less flora than gut (Parasit Vectors 6: 146, 2013).

**Methods:**

Using a metagenomic approach, we have identified for the first time the diverse microbial community associated with these two physiologically different tissues of the digestive system in the mosquito *Anopheles culicifacies*.

**Results:**

A total of 17 different phyla could be assigned to the whole metagenomic dataset, predominated by the phylum Proteobacteria, Firmicutes, Bacteriodetes, Tenericutes and Actinomycetes. Common bacteria included the members of *Enhydrobacter*, *Agromonas*, *Serratia*, *Ralsonia*, *Lactobacillus*, *Pseudomonas*, *Streptococcus*, *Rubrobacter*, *Anaerococcus*, *Methylobacterium*, *Turicibacter*, *Elizabethkingia* etc. in both the tissues representing ‘core microbiota’ of the mosquito digestive system. Salivary associated unique bacterial community included the members of *Chloriflexi*, *Chlorobi*, *Cyanobacteria*, *Nitrospira*, *TM7*, *Armatimonadetes*, *Planctomycetes*, *Fibrobacteres* etc.

**Conclusion:**

We find that the salivary gland microbial community structure is more diverse than gut of the mosquito, probably due to differential feeding associated engagements such as food acquisition, ingestion and digestion processes.

## Background

In their natural habitat, mosquitoes are regularly exposed to several environmentally guided abiotic as well as biotic factors, affecting their reproduction, survival and vector competence [1,2]. The insect gut is believed to be an important interface which not only provides a compatible physiological environment, space and battery of digestive enzymes/proteins to digest diverse nutrients, but also support the growth of gut associated microbial flora [3,4]. Bacterial endosymbionts have now been shown to play many key roles in insect functions such as food digestion, metabolism, reproduction and fighting pathogens [5,6]. In case of the blood feeding insect vectors, especially mosquitoes which transmits medically important infectious diseases e.g. malaria, dengue, filariasis etc., the gut also participates in blood digestion, bacterial proliferation and pathogen development [7]. Despite the fact that adult mosquitoes spend longer time over nectar sugar for regular energy sources; major studies are currently being focused on understanding the gut flora mediated molecular relationship of blood feeding and pathogen transmission [6,8-10]. Surprisingly, insects, especially mosquitoes non-gut tissues of the digestive system e.g. salivary glands also significantly participate in food acquisition, digestion initiation and pathogen transmission [11-15] however, salivary associated bacterial flora have not been investigated in detail. Although some specific bacteria have been shown to be associated with non gut tissues viz. salivary glands, reproductive organs, hemolymph, head, muscles, it is still believed that these organs have fewer microbial flora than gut [1]. Furthermore, there is some experimental evidence indicating that symbiotic microbes provide essential amino acids contributing to the digestion of cellulose in some wood-feeding insects [5] however, we have very limited knowledge exploring the role of sugar feeding associated microbial adaption in mosquitoes.

*Anopheles culicifacies*, an important rural malarial vector transmits more than 65% malaria in India. Current evidence suggests that the intense transmission in the rural areas could be attributed due to its strong adaptation towards agricultural plain areas and complexes of at least five sibling species A, B, C, D, E with wide distribution [16-18]. Due to complex bionomics and limited molecular database knowledge, we are currently engaged to understand the feeding associated molecular and evolutionary complexity of the mosquito *A. culicifacies*. Both salivary gland and gut constitute key partners of the digestive system, but have different structural/functional organization and physiological environments, enabling the salivary gland to initiate salivation, food acquisition, mixing and delivering to the midgut via crop for proper digestion and absorption process [19-21]. Therefore, to gain initial clues on the feeding associated endosymbiotic relationships, in this study we have mapped and compared the microbial community associated with two physiologically different tissues of the same digestive system in the laboratory reared naïve sugar fed mosquito *Anopheles culicifacies*. A comprehensive and comparative metagenomic analysis, has unraveled for the first time that mosquito salivary glands harbor more complex microbial communities than gut, a knowledge which may guide our future investigation to better understand the feeding associated molecular relationships and design vector management strategies.

## Methods

A technical overview and workflow has been presented in the S1 document.

### Mosquito rearing

A cyclic colony of the mosquito *Anopheles culicifacies* sibling species A, were reared and maintained at 28 ± 2°C /RH 80% in the insectary fitted with a simulated dawn and dusk machine, essentially required for proper mating and feeding at NIMR [22]. All protocols used for rearing and maintenance of the mosquito culture were approved by the ethical committee of the institute. For metagenomic analysis, the pupal stage mosquito *Anopheles culicifacies* were collected from the insectary and kept in a round plastic cage fitted with mosquito net, perfectly wiped with 70% ethanol prior to the experiment. Post emergence adult mosquitoes were fed daily on sterile sugar solution (10%) using a glass test tube supplied with a sterile cotton swab throughout the experiment.

### Tissue collection & DNA isolation

For this study we collected salivary glands and gut from 3–4 day old sugar fed adult female mosquitoes. Prior to dissection, mosquitoes were surface sterilized using 70% ethanol for 1 min followed by dissection in saline (1XSTE). Throughout the dissection procedure in the laminar flow, the dissecting stereomicroscope working area was also kept sterilized by using 70% ethanol. Pooled salivary gland (35 pairs) and guts (20 whole gut) were collected into the minimal volume (20ul) of sterile ice cold 1X STE (100 mM NaCl/10 mM Tris Cl, pH 8.0/1 mM EDTA, pH 8.0). Under aseptic conditions, whole DNA was extracted as described previously [23]. Briefly, the tissue was homogenized in 50 ul of STE, followed by proteinase K digestion and centrifugation. Following DNA quality and quantity examination, the DNA samples (120 ng for the salivary gland & 936 ng for the gut) were used for metagenomic analysis. Two amplicon-based 16S rRNA MID tagged libraries were generated for each tissues, by commercial service providers (NxGenBio Life Sciences, New Delhi, India).

### Library sequencing and analysis

For MID tagged libraries, fusion primers FP and RP were used to generate the amplicons covering the variable regions V3 to V6. PCR amplification was performed, using HotStarTaq Plus Master Mix Kit (Qiagen, Valencia, CA) under the following conditions: 94°C for 3 min followed by 32 cycles of 94°C for 30 s; 60°C for 40 s and 72°C for 1 min; and a final elongation step at 72°C for 5 min. All amplicon products from different samples were mixed in equal volumes, and purified using Agencourt Ampure beads (Agencourt Bioscience Corporation, MA, USA. Pooled amplicons were subjected to emulsion PCR (emPCR) followed by bead recovery and bead enrichment, the bead-attached DNAs were denatured with NaOH, and sequencing primers were annealed. A two-region 454 sequencing run was performed on a 70_75 GS PicoTiterPlate (PTP) by using a Genome Sequencer FLX PLUS System (Roche, Nutley, New Jersey) and consecutively sequenced using XLR70 Sequencing Kit according to manufacturer protocol.

The quality filtered raw reads [24], were analyzed using an online version of VAMPS (Visual Analysis of Microbial Population Structure: http://vamps.mbl.edu) pipeline for OTUs creation by Usearch Algorithm [25]. The quality filtered reads were clustered using the combination of ESPRIT, SLP and MOTHUR [26,27]. Initially, this module processed the most abundant sequences (>10 tags) to create first cluster through pairwise distance matrix for less than 0.02, while low abundant sequences (lesser than 10 tags), which are not within a distance of 0.02 are tested against the larger clusters for further addition to the preclusters if possible. The species richness and diversity estimators ACE and Chao1 were calculated using Mothur [26]. The tag distribution frequency was normalized to percentage within the dataset for community visualization, alpha diversity estimation, and relative abundance comparison and rarefaction curve analysis using community visualization tools available on the online version of the VAMP project module. For taxonomic assignment and differential comparison RDP classifier protocol with 80% confidence threshold was followed to define positive identifier using online version2.0 of the RDP project http://rdp.cme.msu.edu[28]. The sequences that could not be assigned bootstrap confidence estimate above the threshold were grouped under ‘unclassified’ taxon.

### PCR based gene expression analysis

The desired tissues viz. salivary glands, midgut were dissected directly in the Trizol. Total RNA was isolated using standard Trizol method, followed by first-strand cDNA synthesis using Random Hexamer primers (Verso cDNA synthesis kit #AB-1453/A, Thermo Scientific). Relative gene expression was assessed by 16S rRNA gene amplification, using SYBR green dye (Biotool Biolabs, Madriad, Spain) in CFX-96 Real-Time PCR machine. PCR cycle parameters involved an initial denaturation at 95°C for 15 min, 40 cycles of 10 s at 94°C, 20 s at 52°C, and 30 s at 72°C. Fluorescence readings were taken at 72°C after each cycle. A final extension at 72°C for 5 min was completed before deriving a melting curve, to confirm the identity of the PCR product. The following sequences: Actin_fw: 5′-TGCGTGACATCAAGGAGAAG-3′/Actin_rev: 5′-GATTCCATACCCAGGAACGA -3′ and 16S_fw: 5′-TCCTACGGGAGGCAGCAGT-3′/16S_rv: 5′-GGACTACCAGGGTATCTAATCCTGTT-3′ were used to design the primers for actin and 16sRNA gene amplification respectively. To better evaluate the relative expression, each experiment was performed in three independent biological replicates. Actin gene was used as an internal control for normalization and Student’s *t-test* was used for statistical analysis.

## Results & discussion

**Figure 1:**
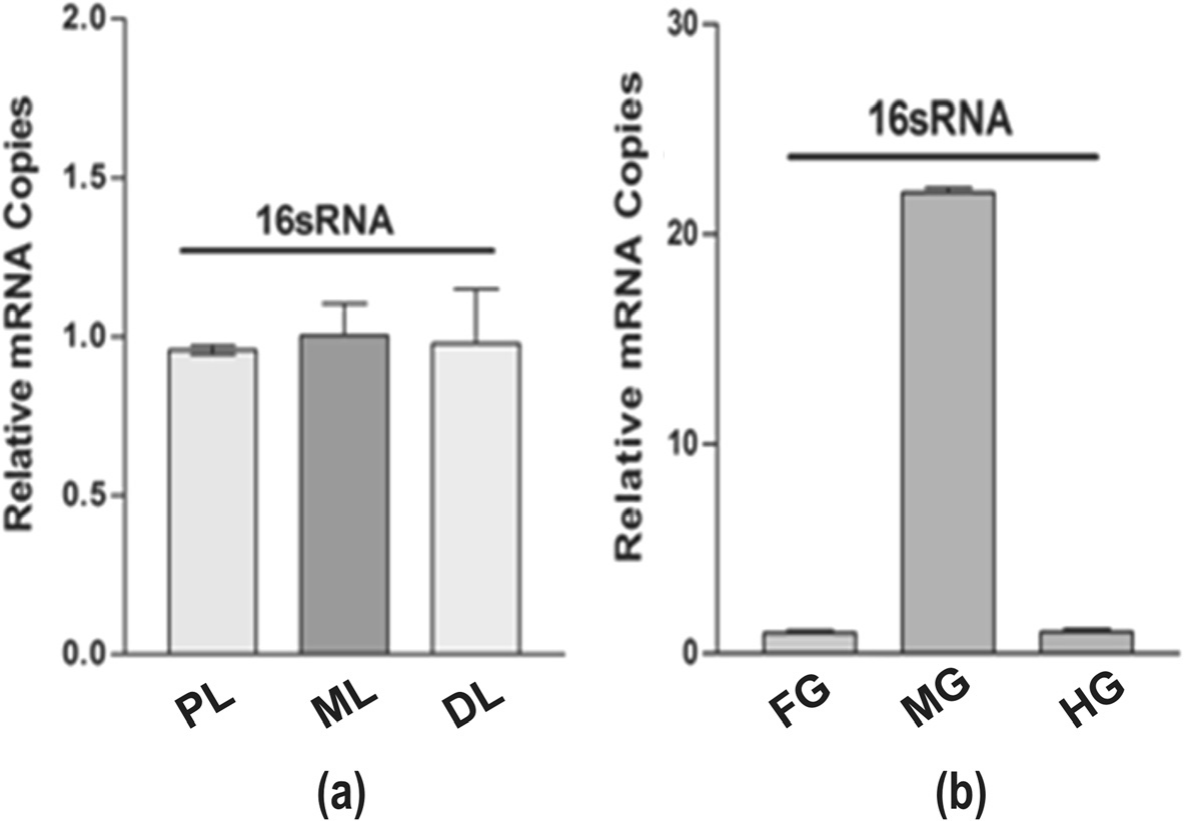
**Tissue specific spatial distribution of bacteria in the digestive system of the mosquito: (a)**. 16sRNA based real time PCR analysis demonstrating the relative expression in the specific lobes viz. Proximal (PL); Medial (ML) and Distal (DL) of the salivary glands. **(b)**. The relative expression of 16sRNA gene in the distinct parts viz. foregut (FG); midgut (MG) and hindgut (HG) of the gut tissue.

Our 16sRNA based real-time PCR analysis indicated that bacteria are equally associated with each of the three salivary lobes (Figure 1a), while predominant in the midgut as compared to the foregut and hind gut of the mosquito digestive system (Figure 1b). Though, the current evidence suggest that mosquito gut associated microbial flora may significantly affect the mosquito genetic factors (e.g. immunity/vector competence) influencing pathogen transmission ability [8,9], there is very limited knowledge in relation to the establishment of symbiotic bacterial communities in different mosquito tissues involved in food digestion. Therefore, to estimate and compare the diversity of the bacterial community, we generated a total of 123325 and 96711 sequence raw reads through pyrosequencing of a 16S rDNA library using 454 Genome Sequencer (Roche, USA) analyzer, for the salivary gland and the gut, respectively (see Additional file 1: work flow-S1). Detailed statistics of the sequencing database is summarized in Table 1.

**Table 1:** Metagemoic library sequencing stat of the salivary and gut tissue of the mosquito digestive system

**S.NO.**	**PARAMETERS**	**Salivary gland**	**Gut**
1	Total number of reads	123325	96711
2	Minimum read length	48	31
3	Maximum read length	1772	869
4	Average read length	598.82	568.98
5	Median read length	757	727
6	Total number of bases	73849474	55027080
7	Total number of HQ bases	68486310	49112801
8	Percentage of HQ bases	92.74%	89.25%
9	Average quality score (Overall)	33.36	29.98
10.	Final High quality reads	(107777/87.39%)	(86367/87.24%)

**Figure 2:**
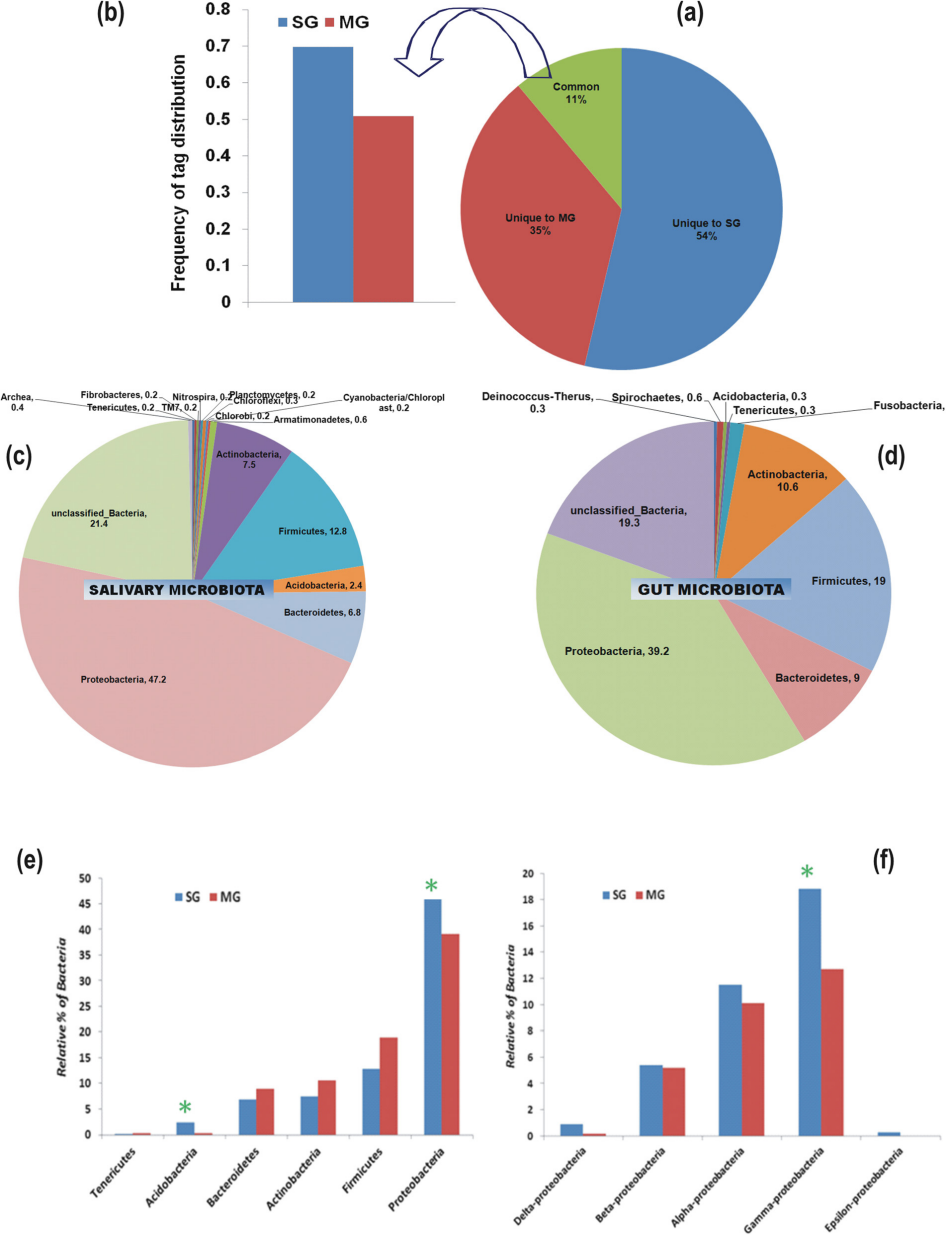
**Taxonomy independent microbial community structure visualization:** Tag distribution frequency (0-100%) analysis was performed to estimate the tissue associated microbial community complexity **(a)**; predominated by the salivary associate microbial community of the overlapped (11%) tags **(b)** as compared to the gut of the mosquito. Microbial flora diversity of two physiologically different tissues of the mosquito digestive system: Taxonomic assignment and relative percentage of the microbial community associated with the salivary glands **(c)** and gut **(d)** in mosquito *A. culicifacies*. The final high quality clustered sequences were independently analyzed through RDP classifier at the phylum level. Dominant Microbial flora community comparison between digestive tissue: To identify and compare the tissue specific microbial communities, both salivary and gut sequence libraries were compared using online RDP classifier program. Bar chart represents the relative percentage of the dominantly associated community classified at Phylum level **(e)** and sub community of the Proteobacteria classified at Class level **(f)**, common to both the tissues. The green * mark indicates the significant difference in the communities at *p-value* (<0.01).

Following quality filtration, total unique tags 2,18,425 that differed by no more than 3% were clustered into 6674 master OTUs dataset and compared to determine the frequency of tag distribution for inter-tissue bacterial diversity structure visualization and analysis (See Additional file 2: supplemental document S2 for complete stat of analysis). Normalized tag distribution frequency (0-100%) analysis revealed unique (54% salivary gland/SG & 35% midgut/MG) as well as overlapped (11%) microbial communities between the two tissues (Figure 2a/Additional file 3: Table S1). Interestingly, the tag distribution frequency of the overlapped microbial community was dominated (Figure 2b) by salivary gland (70%) over the gut (50%). Furthermore, the comparative rarefaction analysis showed the large variability in the bacterial community between the two tissues, covering more taxa counts (OTUs) in the salivary gland (S2). Subsequently, Chao/ACE estimator and multiple α-diversity indices analysis (Table 2) indicated that salivary gland harbors more diverse bacterial flora as compared to the gut in the laboratory reared adult female mosquitoes.

**Table 2:** Tissue specific comparative stat of Bacterial diversity estimation indices

**Sample**	**Sample depth**	**Taxonomy rank**	**Observed richness**	**Chao**	**ACE**	**α-Diversity indices**		
						**Shannon weaver diversity index**	**Simpson diversity index**	**Inverse simpson**
Salivary gland	123316	Class	35	39.5	35.79	1.85	0.77	4.42
Gut	95109	Class	22	22.5	22.2	1.83	0.78	4.49

Different bacterial taxa may influence mosquito physiological and immunological properties e.g. digestion, metabolism and immunity, therefore, we aimed to classify and assign taxa to the level of genera/species. To do this we processed and analyzed our quality filtered data through an online version of RDP Naive Bayesian rRNA Classifier Version 2.5. at default setting of the program (80% confidence threshold) available at 16SrRNA Ribosomal Database Project (See workflow S1). In addition to one Archea (0.4%) associated with salivary gland, a total of 17 different phyla of the bacterial community could be assigned to the whole dataset (Figure 2c,d), predominated by more than 76% of the bacteria belonging to the phylum Proteobacteria, Firmicutes; Bacteriodetes, Tenericutes and Actinomycetes (Figure 2e) while 21.4% (SG) and 19.3% (MG) clusters remains unassigned in both the tissues respectively. However, relative abundance (>0.2%) analysis of the major classes revealed that salivary gland is not only dominated with γ-proteobacteria (P < 0.01) over other common α/β/δ-proteobacteria, but also exclusively harbor ϵ-proteobacteria (0.3%) (Figure 2f). Another phylum Acido-bacteria (Class-Gp1/1.2%; Gp2/0.3%;Gp3/0.4% and Gp4/0.1), also significantly (P < 0.01) dominated in the salivary gland (2.3%) over unclassified Acido-bactetia (0.3%) in the gut (Figure 2e). We also identified other phylums (≥0.2%), uniquely associated either with salivary gland including Armatimonadetes (0.6%); Cyanobacteria/Chloropyta (0.2%); Chlorobi (0.2%); Chlorofelxi (0.3%); Planctomycetes (0.2%); Nitrospira (0.2%); Fibrobacter (0.2%) or the gut including Fusobacteria (1.3%); Deinococcus (0.3%) and Spirochaetes (0.6%) (Figure 2c,d). These findings clearly demonstrated that salivary gland is not only enriched with ‘core microbiota’ but is also uniquely associated with more diverse bacterial taxa than gut.

Lastly, we catalogued the bacteria unevenly distributed in between two tissues, which could be classified at genus level. Common bacteria included the members of the 36 genera predominated by *Enhydrobacter, Agromonas, Serratia, Ralsonia, Lactobacillus, Pseudomonas, Streptococcus, Rubrobacter, Anaerococcus, Methylobacterium, Turicibacter, Elizabethkingia* etc. in both the tissues representing ‘core microbiota’ of the digestive system in mosquito *Anopheles culicifacies* (Additional file 4: Table S2). Interestingly, we did not find any single sequence from the genera of *Asaia* sp., an acetic acid bacteria, which have been dominantly associated with many anopheline mosquito species, including *A. stephensi*[29,30]. Indeed finding of other unique *Enhydrobacter*- γ-proteobacteria (MG-3.5%/SG-5.2%) and *Agromonas*- α-proteobacteria (MG-1.6%/SG-1.6%), *Rubrobacter*-Actinobacteria (MG-0.5%/SG-0.8%), *Turicibacter*-Firmicutes (MG-0.2%/SG-0.5%), dominantly associated with both the tissues in the mosquito *A. culicifacies*, provide initial evidence that symbiotic association of microbial communities may favor ecological adaptation of specific mosquito species to the diverse nutrient sources. Although, it is also important to note that both *A. stephensi* as well as *A. culicifacies* are primary malarial vectors in India, but prefer different ecological habitats. *A. stephensi* is an urban malarial vector, whereas mosquito *Anopheles culicifacies* prefer adaptation over plain agricultural areas of rural India [16]. This could be one of the possible explanations for the missing *Asaia* sp. from *A. culicifacies*. However, further investigations are required to validate these propositions in different mosquito species. Furthermore, we also identify and classify the sequences related to genus unique to salivary gland (total 76) or the midgut (total 46), unraveling a tissue specific diversified microbial community (Additional file 4: Table S2).

Although our knowledge for the role of genus *Enhydrobacter* (Gram-negative)-γ-proteobacteria, *Agromonas* (Gram-negative)-α-proteobacteria and Acido-bacteria (Gram-negative) association in mosquito is very limited, further characterization of these dominating unique symbionts, could allow us to understand the feeding associated benefits to the mosquito. For example, the dominant occurrence of *Acidobacteria* spp. in the gut of the wood feeding larvae of Huhu Beetle (*Prionoplus reticulari*), suggests that these bacteria may facilitate the degradation and metabolization of highly polymerized diverse plant sugars [31]. More recently, the finding of several unique bacterial communities viz. *Chloriflexi, Chlorobi, Cyanobacteria, Nitrospira, TM7, Spirochaetes, Fusobacteria, Enhydrobacter* (which we also find in the present study), associated with the gut of the mosquito *Culex tarsalis* larvae, reared in different wet land habitats [32], suggest that such diverse microbes could significantly influence the feeding and adaptation of the mosquitoes to different ecological habitats. The *Agromonas*, previously isolated from paddy fields [33], belongs to soild oligotrophs (nitrogen fixing bacteria), usually grown at extra low nutrient environments, and has remained unidentified from any insect species so far.

## Conclusion

In summary, we find that the salivary gland microbial community structure is more diverse than gut of the mosquito, probably due to differential feeding associated engagements such as food acquisition, ingestion and digestion processes. This knowledge may guide our future investigation to better understand the feeding associated molecular relationships and design vector management strategies.

## Competing interest

The authors declare that they have no competing interests.

## Authors’ contributions

Conceived and designed the experiments: PS, RD, NV, NS, KCP. Performed the experiments: PS, TDD, TT, SL. Analyzed the data: RD, RKM, PS, SS. Contributed reagents/materials/analysis tools: RD, NV, KCP. Wrote the paper: RD, PS, NV, KCP. All authors read and approved the final manuscript.

## Authors’ information

The sequence data has been submitted to NCBI SRA database under following accession number: ACA-SF_SG:SRR1017625 & ACA-SF_MG: SRR1017626. There is no competing financial interest to declare. Correspondence and request for material should be addressed to RD (rkd1976.rajnikant@gmail.com).

## Supplementary Material

Additional file 1 S1: Work Flow (Metagenomics). Click here for file

Additional file 2 **S2: Details stat of tissue specific comparative analysis:** Tag distribution/frequency map analysis and comparison: For the relative abundance and microbial diversity analysis the whole dataset was analyzed using online accessible program available at Visualization and Analysis of Microbial Population Structure (VAMP Project) (http://vamps.mbl.edu/). **(a)** Frequency heat map at Phylum level; **(b)** tissue specific Pie chart analysis for frequency mapping (0-100%) at class level; the relative complexity of the microbial community by Rarefaction curve analysis **(c)**; and taxonomic rank abundance for salivary glands **(d)** and gut **(e)**. Click here for file

Additional file 3: Table S1 Detailed OUT output and data analysis. Click here for file

Additional file 4: Table S2 Tissue specific unique bacteria classified at Genus. Click here for file
