# Supplementary figures and images for: Salivary glands harbor more diverse microbial communities than gut in Anopheles culicifacies
Source: Parasit Vectors. 2014 May 20;7:235. doi: 10.1186/1756-3305-7-235 (PMC4062515; doi:10.1186/1756-3305-7-235)

## S1: Work Flow (Metagenomics)

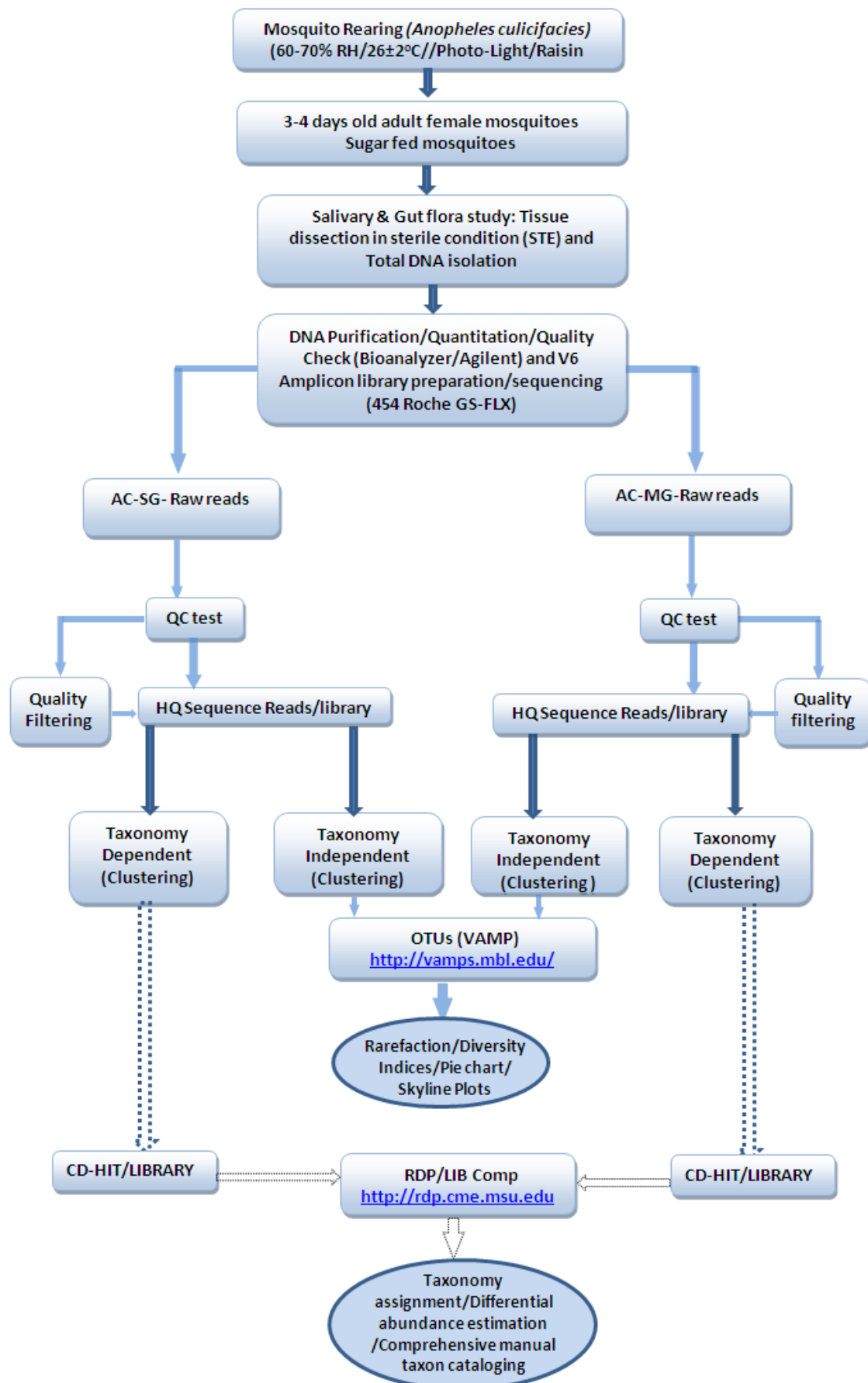

Supplement: Additional file 1 — S1: Work Flow (Metagenomics). [file 1756-3305-7-235-S1.pdf]
