# Supplementary material for: Salivary glands harbor more diverse microbial communities than gut in Anopheles culicifacies
Source: Parasit Vectors. 2014 May 20;7:235. doi: 10.1186/1756-3305-7-235 (PMC4062515; doi:10.1186/1756-3305-7-235)
Supplement: Additional file 2 — S2: Details stat of tissue specific comparative analysis: Tag distribution/frequency map analysis and comparison: For the relative abundance and microbial diversity analysis the whole dataset was analyzed using online accessible program available at Visualization and Analysis of Microbial Population Structure (VAMP Project) (http://vamps.mbl.edu/). (a) Frequency heat map at Phylum level; (b) tissue specific Pie chart analysis for frequency mapping (0-100%) at class level; the relative complexity of the microbial community by Rarefaction curve analysis (c); and taxonomic rank abundance for salivary glands (d) and gut (e). [file 1756-3305-7-235-S2.docx]

**S3: Details stat of tissue specific comparative analysis:**

**Tag distribution/frequency map analysis and comparison:**  For the relative abundance and microbial diversity analysis the whole dataset was analyzed using online accessible program available at Visualization and Analysis of Microbial Population Structure (VAMP Project) (<http://vamps.mbl.edu/>). **(a)** Frequency heat map at Phylum level; **(b)** tissue specific Pie chart analysis for frequency mapping (0-100%) at class level; the relative complexity of the microbial community by Rarefaction curve analysis **(c);** and taxonomic rank abundance for salivary glands (**d)** and gut **(e)**

1.
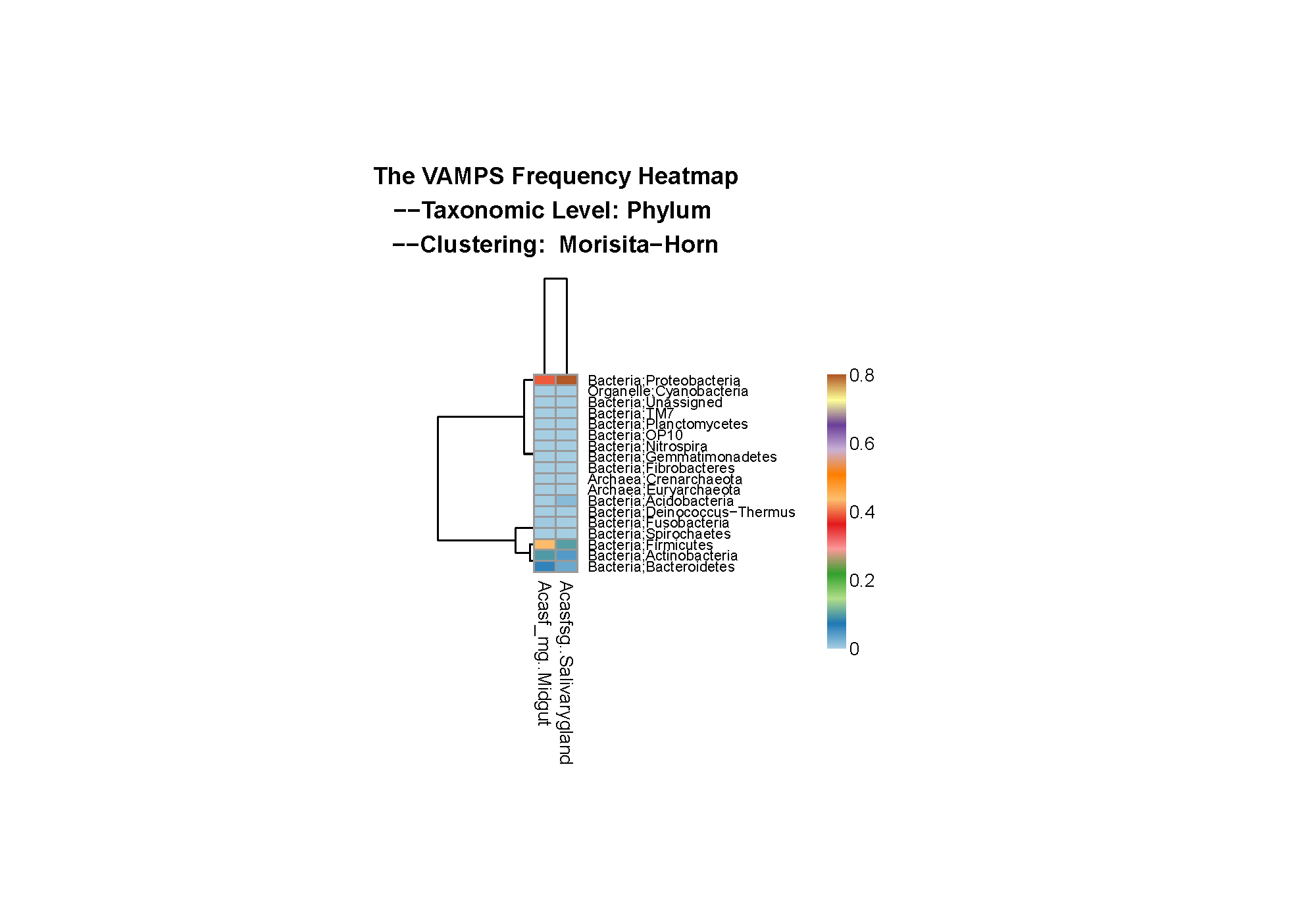


**(a)**


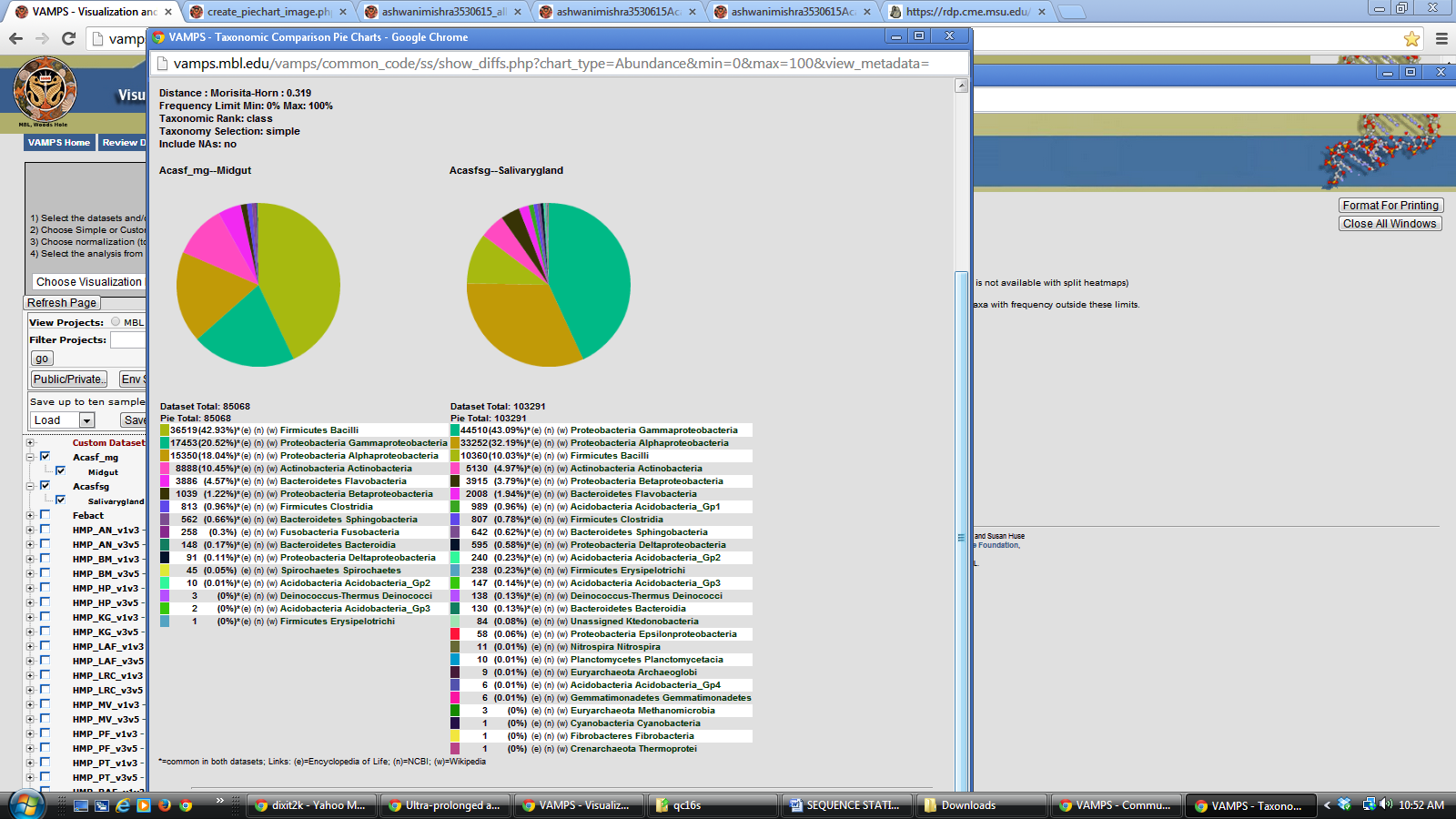


**(b)**


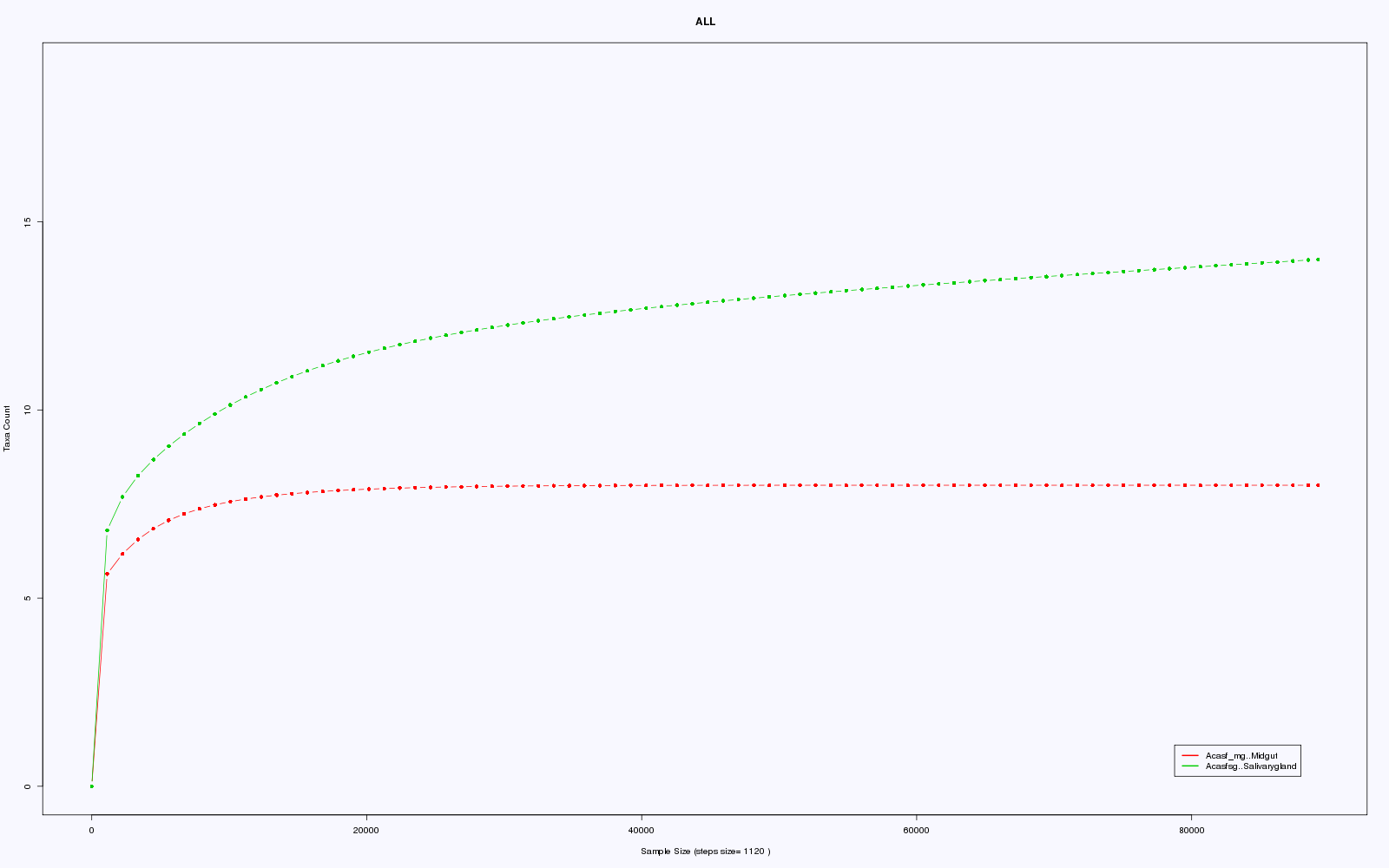


**(c)**


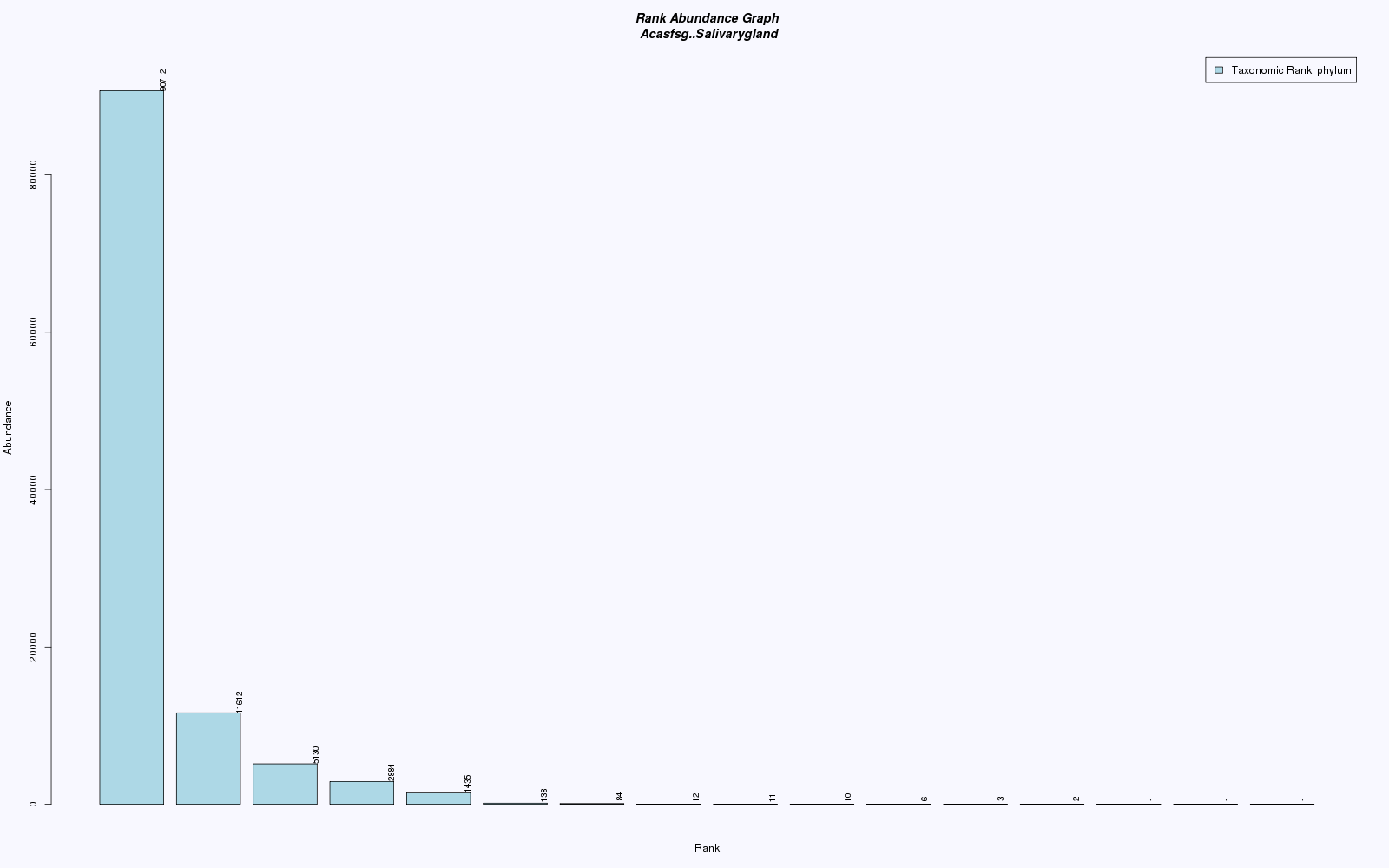


**(d)**


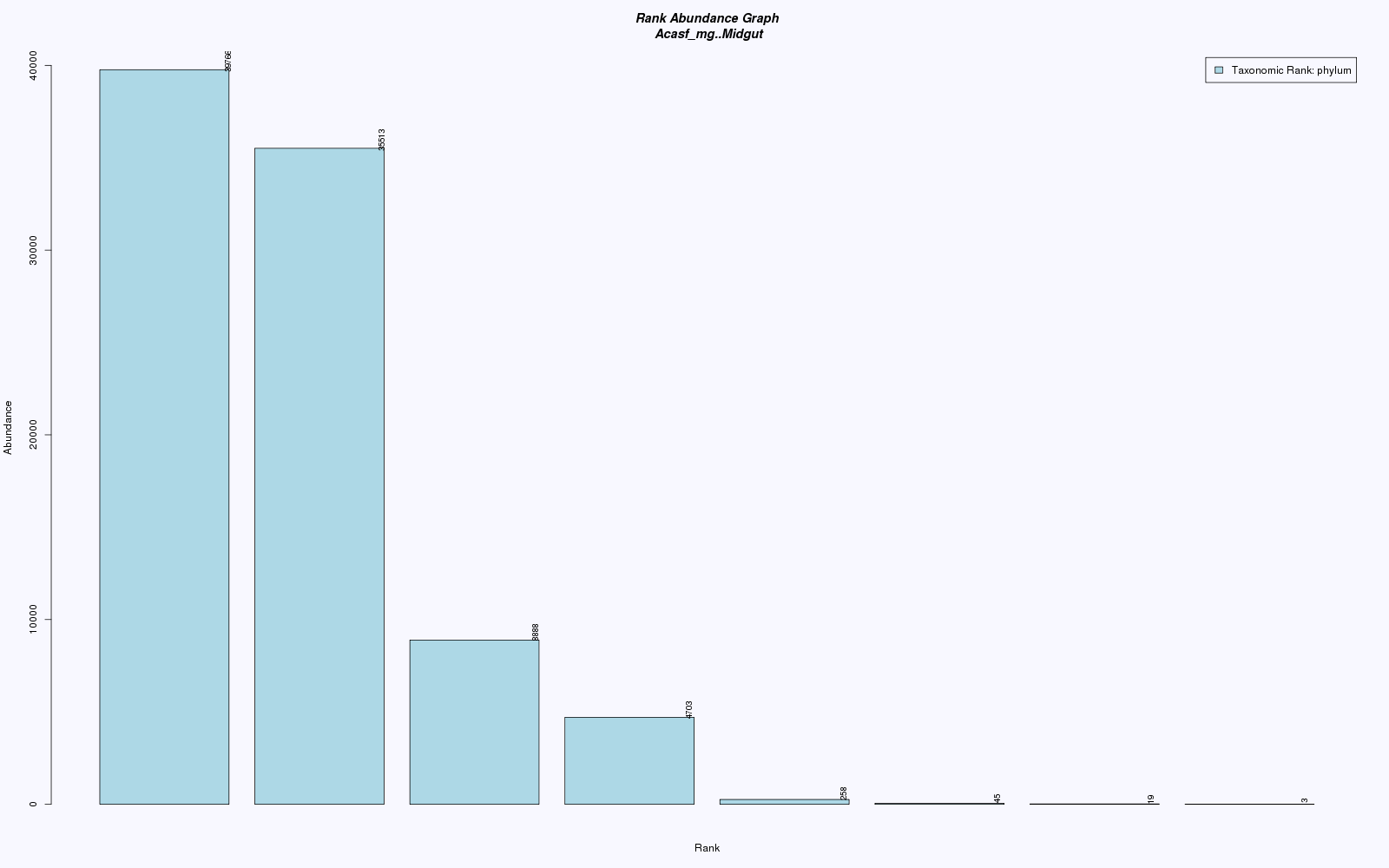


**(e)**
